# Association of COVID-19 Vaccination With Influenza Vaccine History and Changes in Influenza Vaccination

**DOI:** 10.1001/jamanetworkopen.2022.41888

**Published:** 2022-11-14

**Authors:** Andrew M. Parker, Samer Atshan, Matthew M. Walsh, Courtney A. Gidengil, Raffaele Vardavas

**Affiliations:** 1 RAND Corporation, Pittsburgh, Pennsylvania; 2 Pardee RAND Graduate School, Santa Monica, California; 3 RAND Corporation, Boston, Massachusetts; 4 RAND Corporation, Santa Monica, California

## Abstract

This survey study assesses how COVID-19 vaccination differs across historical influenza vaccination patterns and whether influenza vaccination changed during the COVID-19 pandemic.

## Introduction

Understanding willingness to receive new vaccines is critical for vaccine rollout and addressing vaccine hesitancy.^1^ Future COVID-19 and influenza vaccination may coincide, creating a need to understand the dynamics between ongoing vaccine adherence for familiar diseases and novel vaccine acceptance. We combine longitudinal data with a classification of individual influenza vaccination histories^2^ to answer 2 questions: (1) how COVID-19 vaccination differs across historical influenza vaccination patterns, and (2) how influenza vaccination changed during the COVID-19 pandemic.

## Methods

This survey study’s longitudinal survey data came from RAND’s American Life Panel (ALP), a probability-sampled panel of US adults. Walsh and colleagues^2^ analyzed ALP self-reported vaccination across 6 influenza seasons spanning 2009 to 2017. Modeling individuals’ year-to-year tendency to repeat prior vaccination behaviors, panelists were classified as never, sometimes, or always influenza vaccinators.^2^ Subsequent influenza vaccination behavior (2019 to 2020) was assessed in May to June 2020 (N = 1643; 85% completion), which was largely prepandemic. COVID-19 vaccination, along with 2020 to 2021 and 2021 to 2022 influenza vaccination, was assessed in February to March 2022 (N = 2145; 68.8% completion). COVID-19 vaccination is operationalized as fully vaccinated (primary series, meaning single dose Janssen/Johnson & Johnson or 2 doses Pfizer/BioNTech or Moderna) vs not; boosters are not considered. Race and ethnicity were self-reported. An analytic sample of 1366 respondents have complete data. Sampling weights were applied for all analyses. Recruitment and retention are detailed in the eMethods of the Supplement. Online consent was obtained in accordance with study approval by RAND’s Human Subjects Protection Committee. Our approach followed the (AAPOR) reporting guideline. Two-sided *P* < .05 was considered statistically significant. Analyses were performed from June to September 2022.

## Results

Among the 1366 survey respondents, 771 (56%) were female, 33 (2%) were Asian, 101 (7%) were Black, 156 (11%) were Hispanic, and 1161 (85%) were White; mean (SD) age was 56 (13) years. Table 1 describes how historical influenza vaccination classification was associated with later influenza and COVID-19 vaccination. For example, 81.4% (291 of 358) to 92.2% (330 of 358) of individuals classified as always influenza vaccinators through 2017 continued to vaccinate against influenza 2 to 4 influenza seasons later. Conversely, 20.3% (130 of 642) of individuals classified as never influenza vaccinators through 2017 got influenza vaccination in 2019-2020, which was largely prepandemic; this increased to 23.5% (151 of 642) during the pandemic. Individuals classified as always influenza vaccinators were 24.7% more likely to get COVID-19 vaccination vs never influenza vaccinators.

**Table 1.** Proportion Receiving Influenza and COVID-19 Vaccines by Individual Influenza Vaccination History

	Individual influenza vaccination history (2009-2017), No. (%)		
	Never vaccinate	Sometimes vaccinate	Always vaccinate
Vaccine received, No.	642	367	358
2019-20 influenza	130 (20.3)	253 (68.9)	330 (92.2)
2020-21 influenza	151 (23.5)	233 (63.7)	312 (87.2)
2021-22 influenza^a^	147 (22.9)	205 (56.0)	291 (81.4)
Primary COVID-19 vaccine series^a^	413 (64.3)	294 (80.2)	319 (89.0)

^a^ As of February-March 2022.

Overall, the probability of full COVID-19 vaccination was 50% higher if the respondent reported getting the influenza vaccine in the 2021-2022 season (90.9% [858 of 944] vs 60.9% [440 of 723]; risk ratio [RR], 1.50; 95% CI, 1.40-1.59). Conversely, 2021-2022 influenza vaccination was 230% higher if the respondent reported getting a full initial COVID-19 vaccine (57.1% [585 of 1025] vs 17.3% [59 of 341]; RR, 3.30 [95% CI, 2.65-4.27]).

Table 2 focuses only on panelists classified as never influenza vaccinators through 2017. Those receiving the COVID-19 vaccine were significantly more likely to have switched from not receiving (in 2020) to receiving (in 2022) the influenza vaccine (OR, 12.82; 95% CI, 1.46-112.67). Both outcomes were more likely among more educated. Identifying as Democrat (vs Republican) was associated with COVID-19 vaccination (OR, 4.43; 95% CI, 1.51-12.97), but not associated with switching from no influenza vaccination to influenza vaccination.

**Table 2.** Weighted Logistic Regressions Estimating COVID-19 Vaccination and Change in Influenza Vaccination During the Pandemic Among Those Who Historically Were Never Vaccinated

Variables	OR (95% CI)	
	Model 1: Received full primary series of COVID-19 vaccine	Model 2: Received flu shot in 2022 but not in 2020
Received 2021-2022 flu vaccine	4.26 (0.72-25.16)	NA
Received 2020-2021 flu vaccine	1.76 (0.36-8.56)	NA
Received 2019-2020 flu vaccine	1.04 (0.21-5.25)	NA
Received full primary series of COVID-19 vaccine	NA	12.82 (1.46-112.67)
Female	0.44 (0.21-0.92)	1.89 (0.71-5.02)
Age	1.02 (1.00-1.05)	1.03 (1.00-1.06)
Married	2.19 (0.85-5.62)	3.95 (1.04-14.95)
Urban	1.77 (0.77-4.09)	0.54 (0.20-1.49)
Hispanic	0.93 (0.34-2.57)	4.84 (1.05-22.26)
Race		
White	[Reference]	[Reference]
Asian^a^	NA	0.08 (0.01-0.64)
Black	0.72 (0.22-2.32)	0.44 (0.11-1.78)
Other^b^	1.54 (0.14-16.85)	0.04 (0.00-0.36)
Education		
High school or less	[Reference]	[Reference]
Some college	1.04 (0.40-2.69)	0.64 (0.13-3.24)
BS or more	6.46 (2.79-14.94)	3.72 (1.07-12.91)
Unemployed	1.00 (0.24-4.18)	11.42 (1.81-72.09)
Political affiliation		
Republican	[Reference]	[Reference]
Democrat	4.43 (1.51-12.97)	1.65 (0.59-4.58)
Independent	2.19 (0.89-5.38)	2.33 (0.64-8.52)
Other	4.55 (0.85-24.38)	24.70 (4.43-137.84)
Not sure	1.40 (0.42-4.69)	1.49 (0.15-14.84)

Abbreviations: OR, odds ratio.

^a^ Coefficient on Asian race category was not defined because all Asian individuals in the sample received full primary series of the COVID-19 vaccine.

^b^ Includes American Indian or Alaska Native and self-reported other.

## Discussion

This survey study uses a historical data set that classifies influenza vaccination behavior across 8 years,^2^ following these same individuals up to 5 years later. The results of this study are limited by self-reported vaccination, the validity of which is supported by past research.^3^

COVID-19 vaccination was highest among those who historically always received the influenza vaccine, reinforcing studies showing shorter-term correlation between influenza and COVID-19 vaccination.^4,5^ Most strikingly, among individuals who historically never got the influenza vaccine, those receiving COVID-19 vaccine were substantially more likely to switch toward getting the influenza vaccine. This suggests that investing in vaccine acceptance has payoffs beyond the vaccine itself.

## Notes

Supplement. **eMethods.** Supplementary Materials
