# Supplementary material for: Association of COVID-19 Vaccination With Influenza Vaccine History and Changes in Influenza Vaccination
Source: JAMA Netw Open. 2022 Nov 14;5(11):e2241888. doi: 10.1001/jamanetworkopen.2022.41888 (PMC9664264; doi:10.1001/jamanetworkopen.2022.41888)
Supplement: Supplement. — eMethods. Supplementary Materials [file jamanetwopen-e2241888-s001.pdf]

Supplemental Online Content

Parker AM, Atshan S, Walsh MM, Gidengil CA, Vardavas R. Association of COVID-19 vaccination with influenza vaccine history and changes in influenza vaccination. *JAMA Netw Open.* 2022;5(11):e2241888. doi:10.1001/jamanetworkopen.2022.41888

eMethods. Supplementary Materials

This supplemental material has been provided by the authors to give readers additional information about their work.

© 2022 Parker AM et al. *JAMA Network Open.*

eMethods. Supplementary Materials

Respondents come from an ongoing longitudinal survey on the RAND American Life Panel (ALP). Initially, 2179 ALP panelists (84% completion amongst panelists invited to participate) were recruited in 2016, with surveys twice a year for four years (through Summer of 2020). Influenza vaccination for the prior season was assessed each Spring-Summer. Walsh et al (2020) combined data from the first year of this study (2016-2017) with prior ALP surveys to classify ALP panelists as never, sometimes, or always influenza vaccinators. The 2019-2020 influenza vaccination data were from the eighth and final wave of this study (n=1643, 75% of the initial 2179).

A follow-on study recruited all participants in the prior study that remained in the ALP, along with a refresh subsample not considered here. COVID-19 vaccination and 2020-2021 and 2021-2022 influenza vaccination data come from the first survey wave in this follow-on study (n=1405, 64.4% retention). Table S1 breaks down the original 2179 sample, comparing those in and not in the current analytic sample.

© 2022 Parker AM et al. *JAMA Network Open.*

Table S1. Demographic comparison of original 2016 respondents retained and not retained in the analytic sample.

Characteristic	Retained No. (%)	Not Retained No. (%)	p-value[1]
Subsample size	1,366	813	
NSA Status			0.27
Never	497 (36.4)	322 (39.9)	
Sometimes	447 (32.7)	246 (30.4)	
Always	422 (30.9)	241 (29.8)	
Unknown	0	4	
Sex			0.95
Male	595 (43.6)	353 (43.5)	
Female	771 (56.4)	460 (56.6)	
Age, Mean (SD)	56 (13.0)	56 (15.6)	0.90
Married	852 (62.4)	448 (55.1)	<0.001
Hispanic	156 (11.4)	151 (18.6)	<0.001
Race			<0.001
Asian[2]	33 (2.4)	22 (2.7)	
Black	101 (7.4)	95 (11.7)	
White	1,161 (85.1)	623 (76.6)	
Other	70 (5.1)	73 (9.0)	
Unknown	1	0	
Education			<0.001
HS or Less	177 (12.9)	166 (20.4)	
Some College	490 (35.9)	316 (38.9)	
BS or more	699 (51.2)	331 (40.7)	
Unemployed	55 (4.0)	41 (5.0)	0.26

[1] Bivariate comparisons using Pearson's Chi-squared tests (categorical variables) and Wilcoxon rank sum test (Age)
[2] Includes American Indian or Alaskan Native and self-reported “Other”.

© 2022 Parker AM et al. *JAMA Network Open.*
